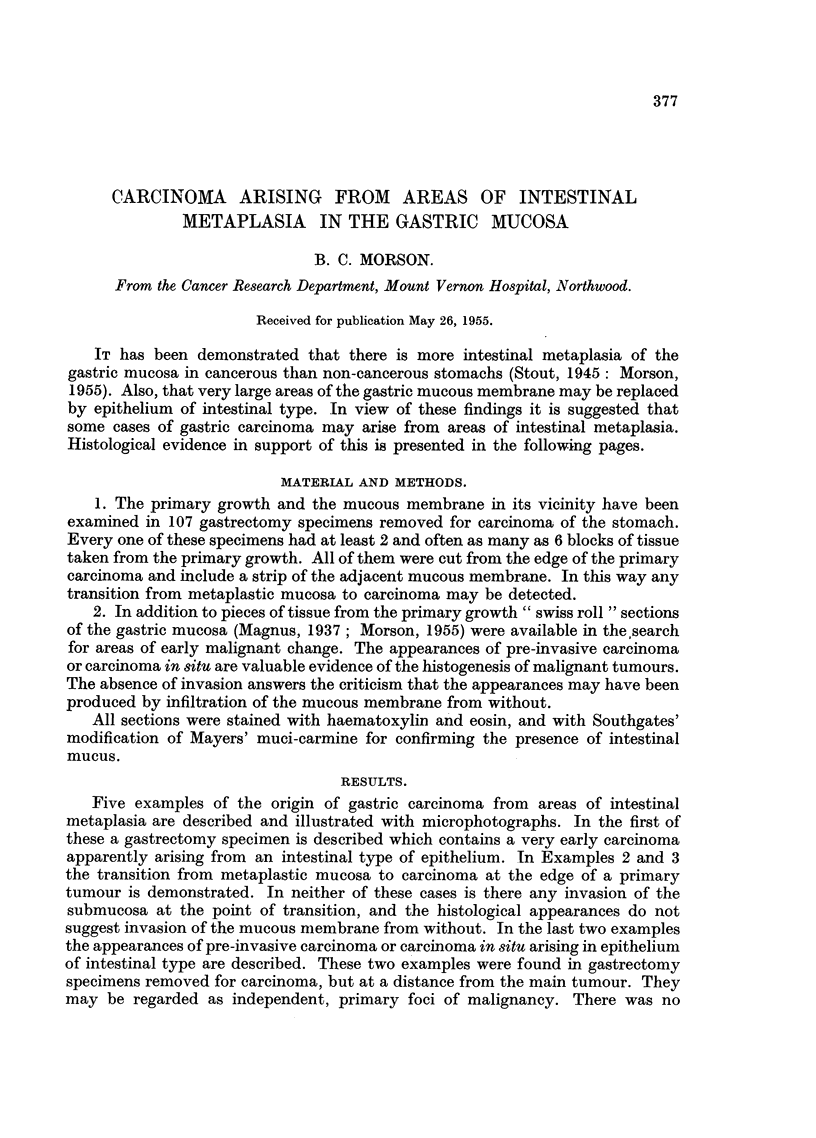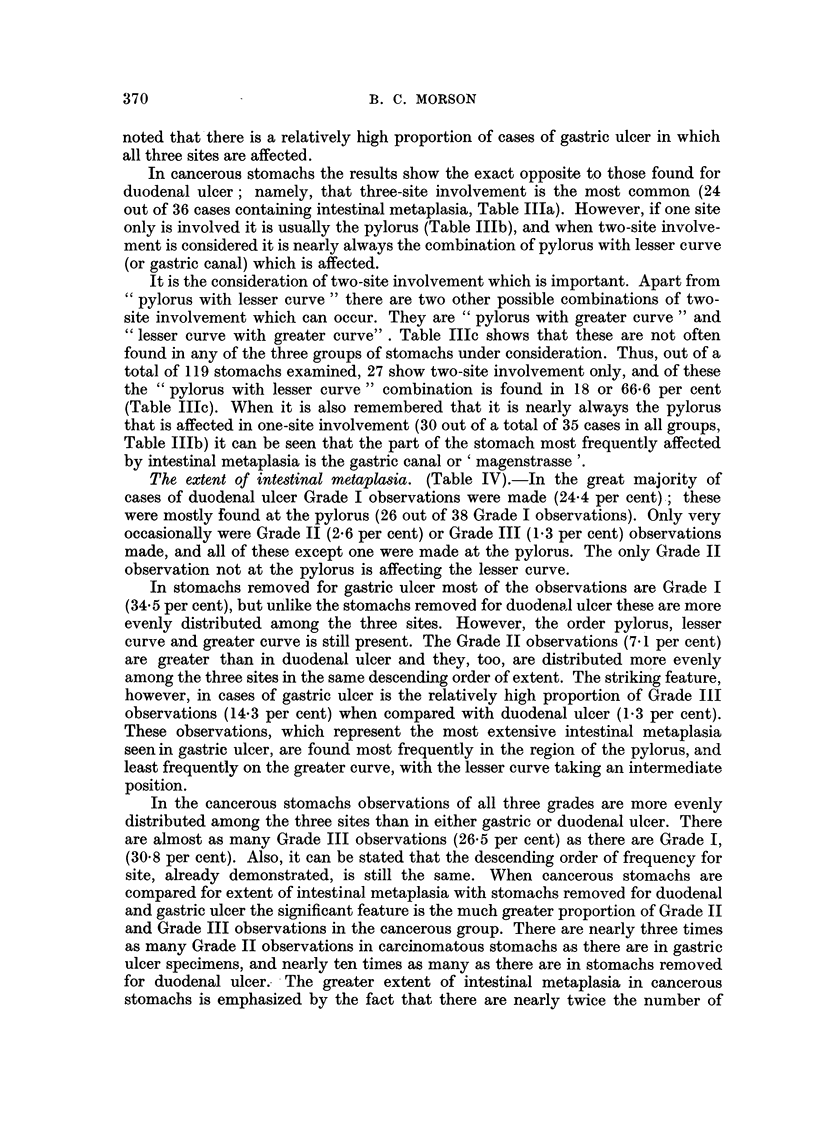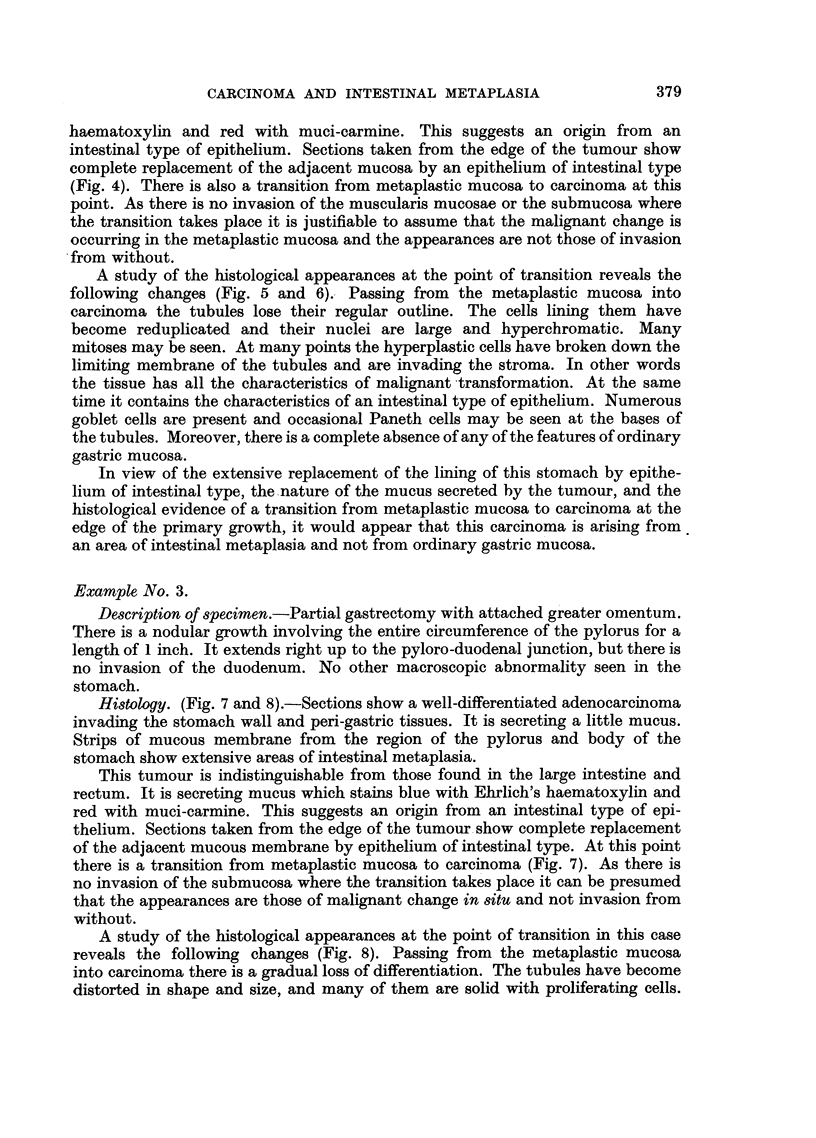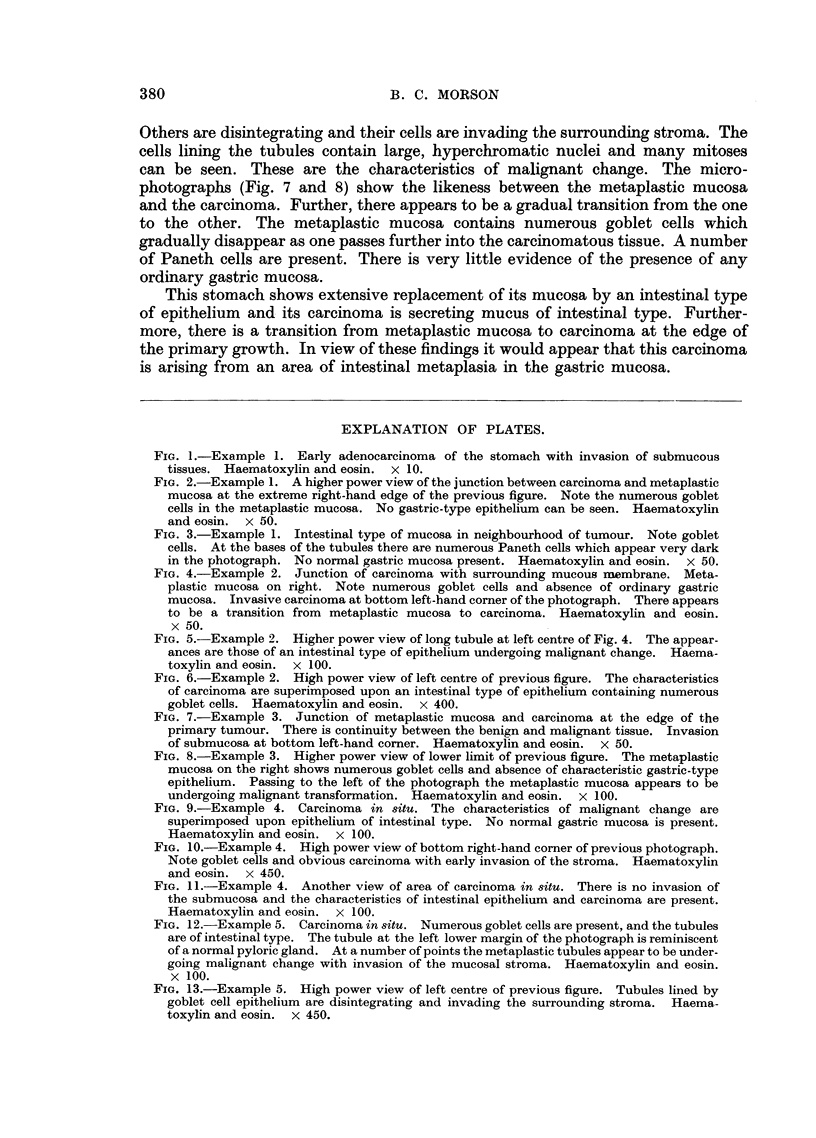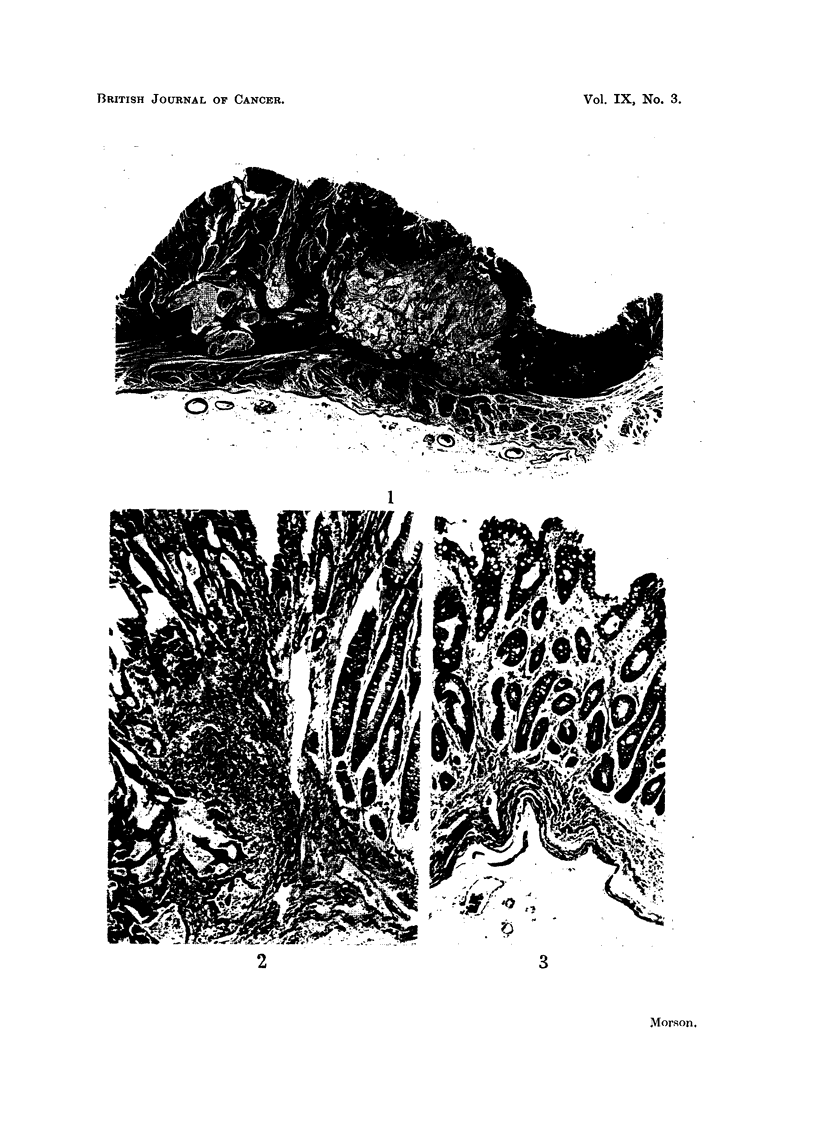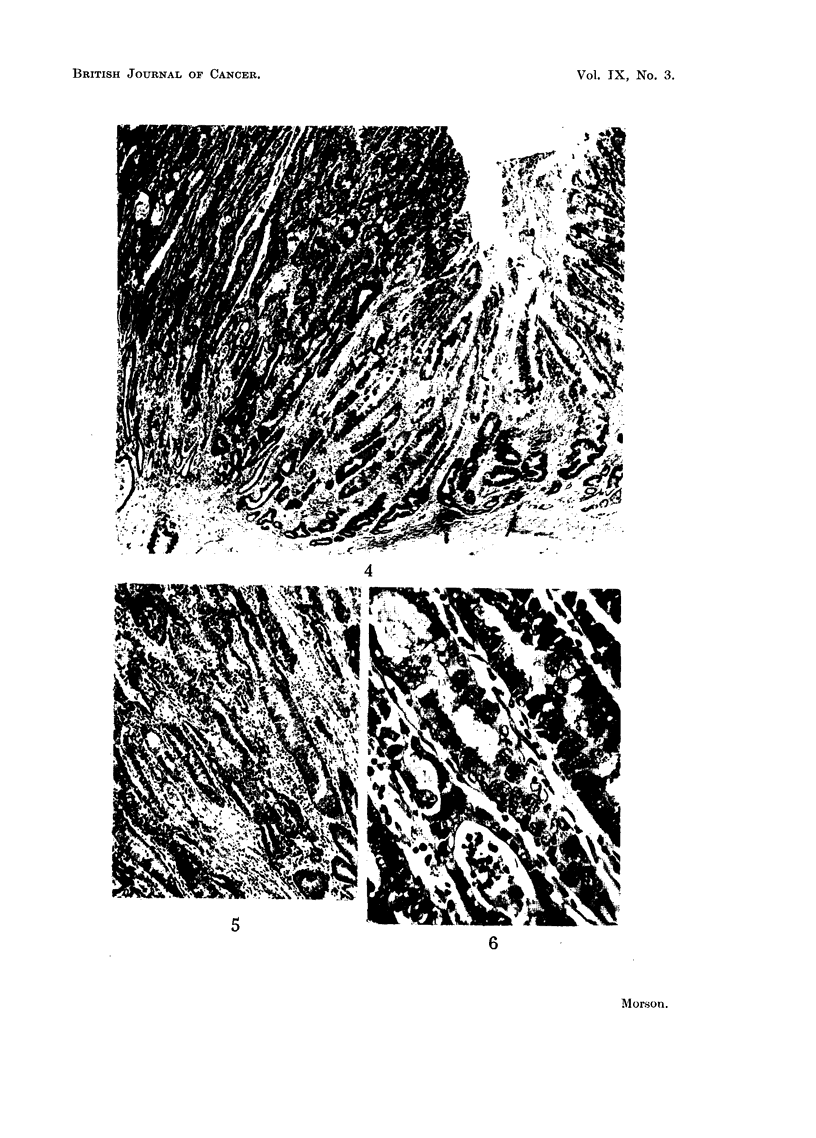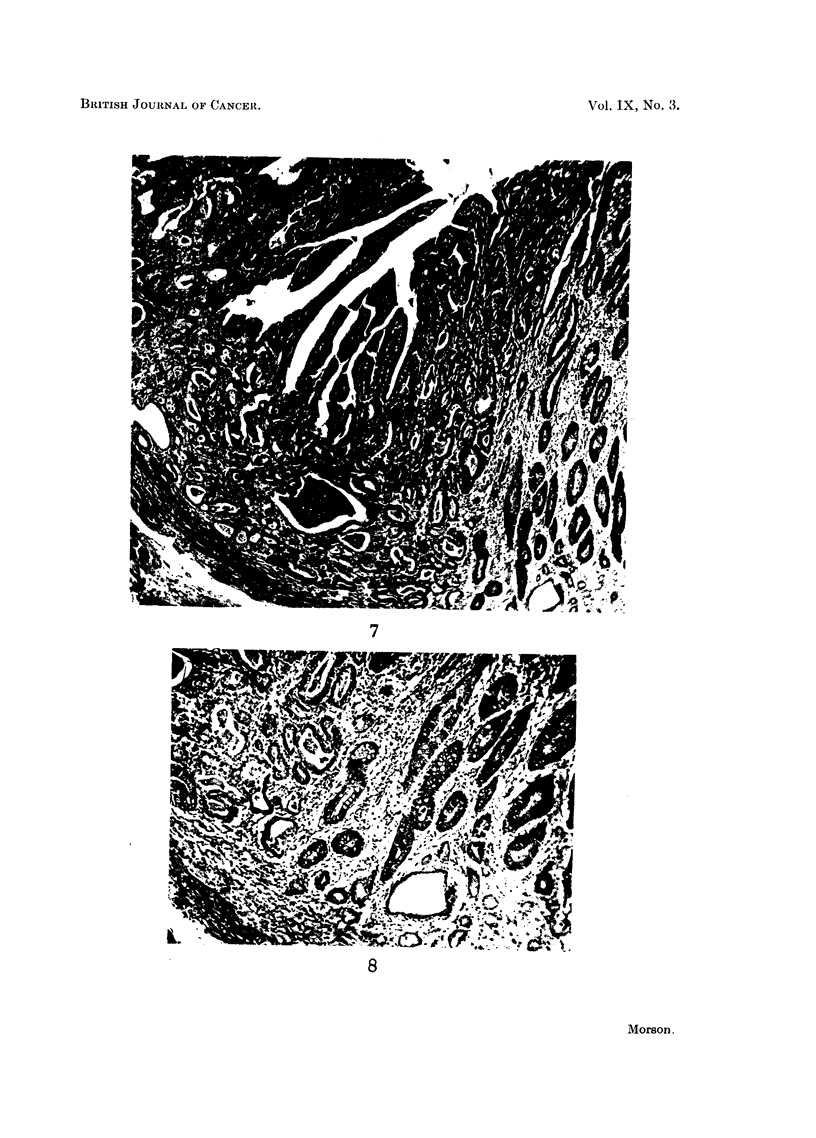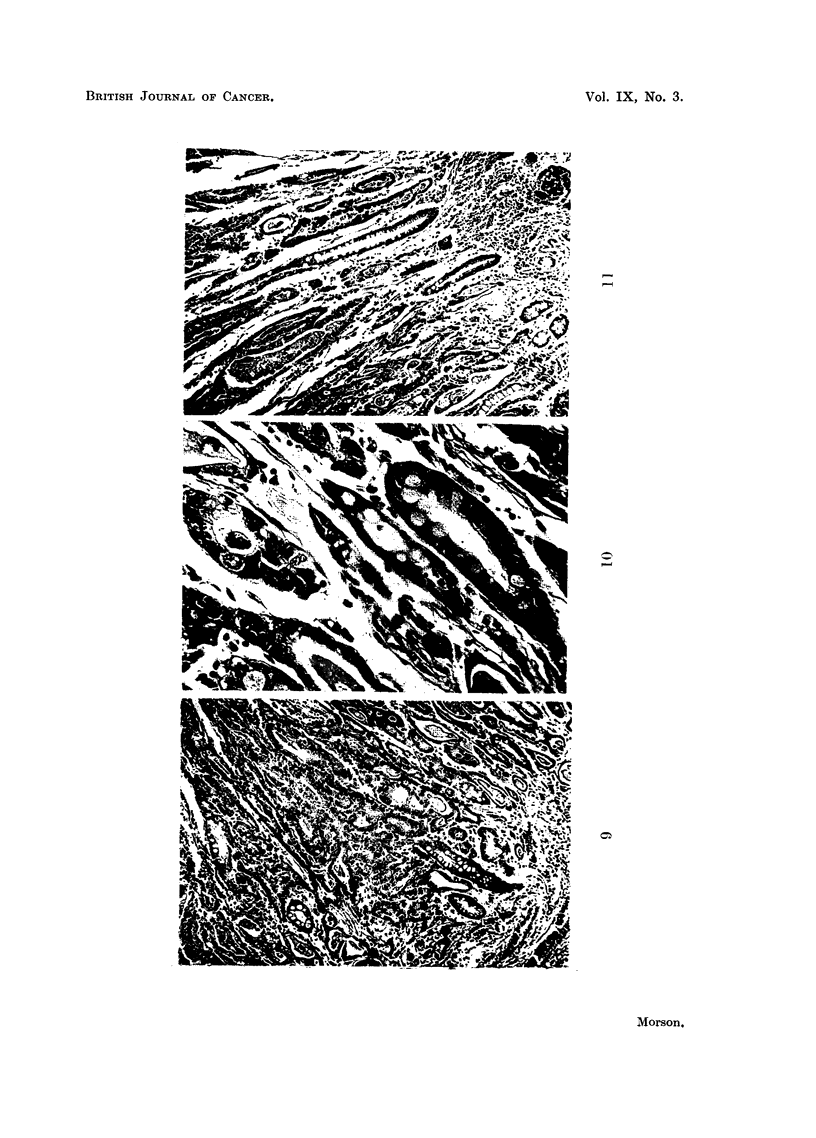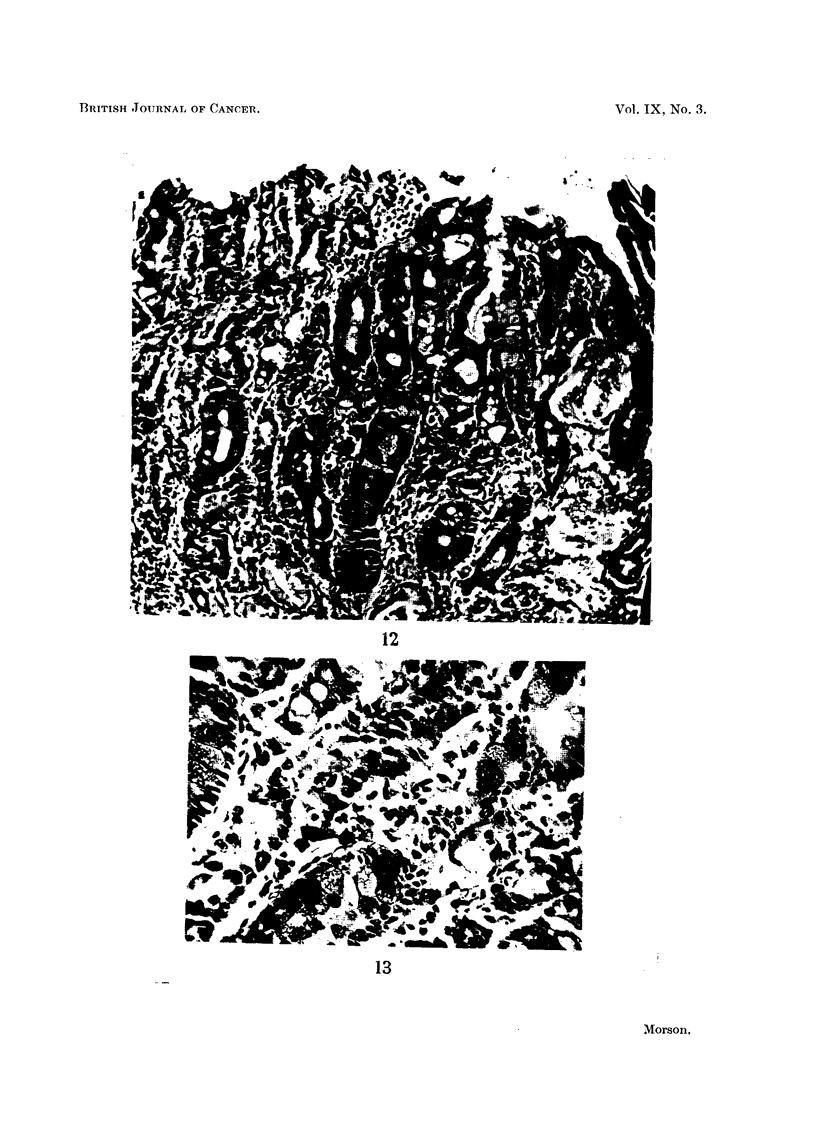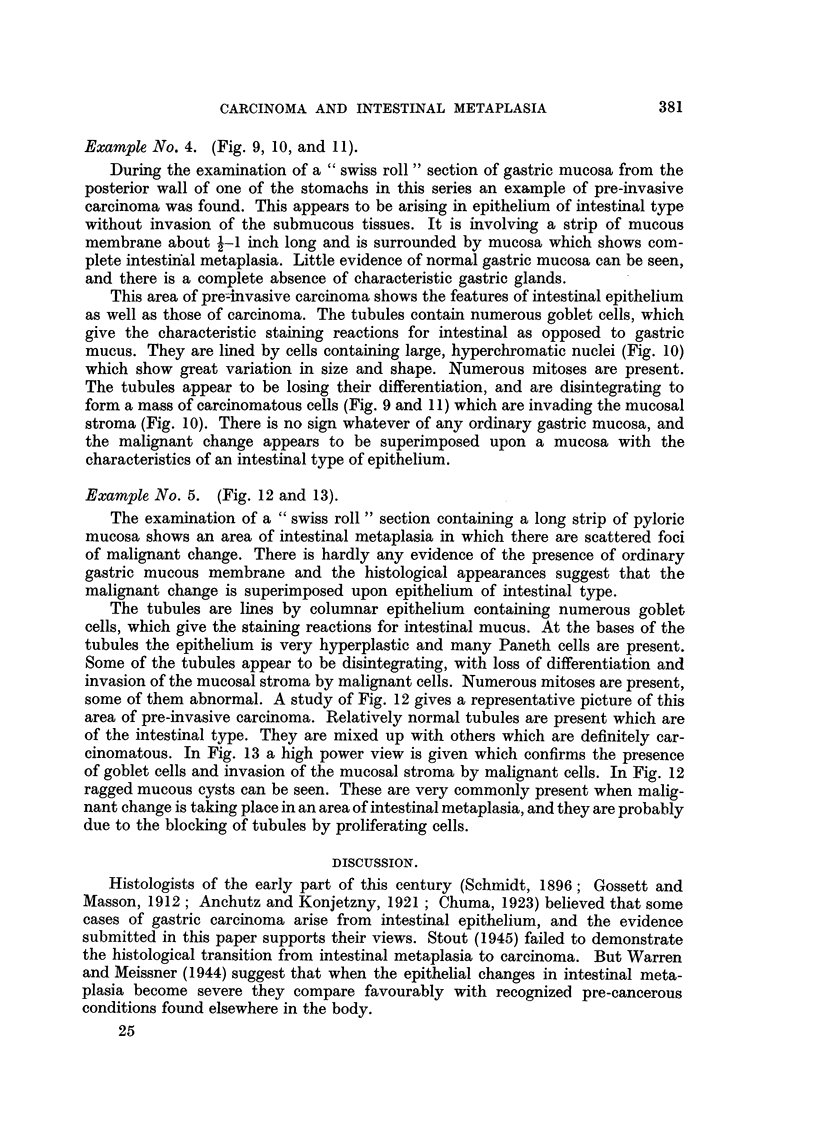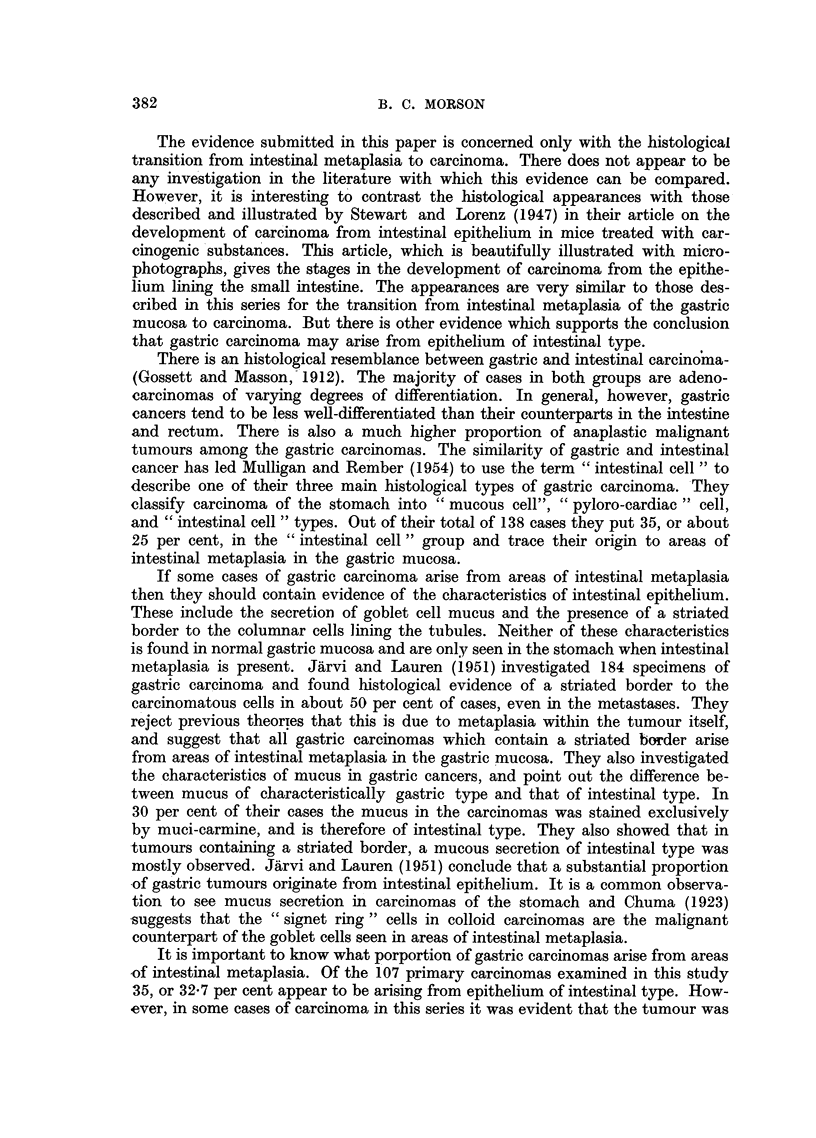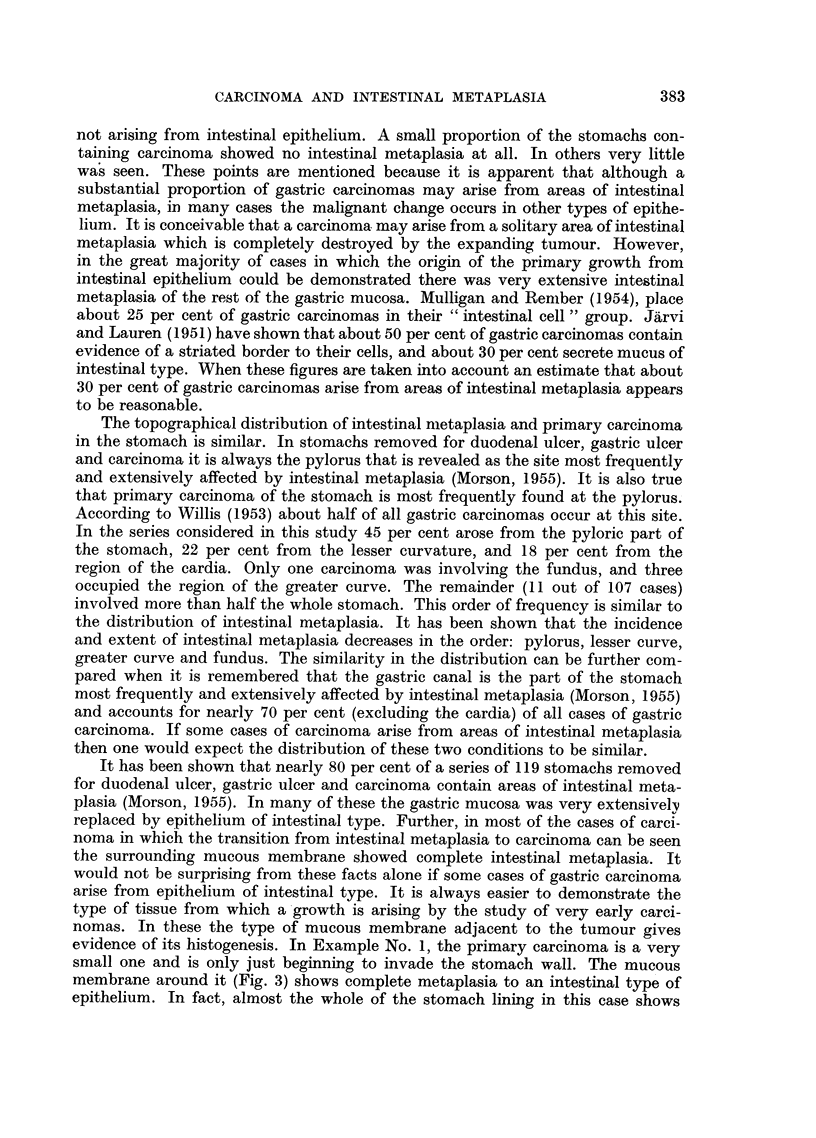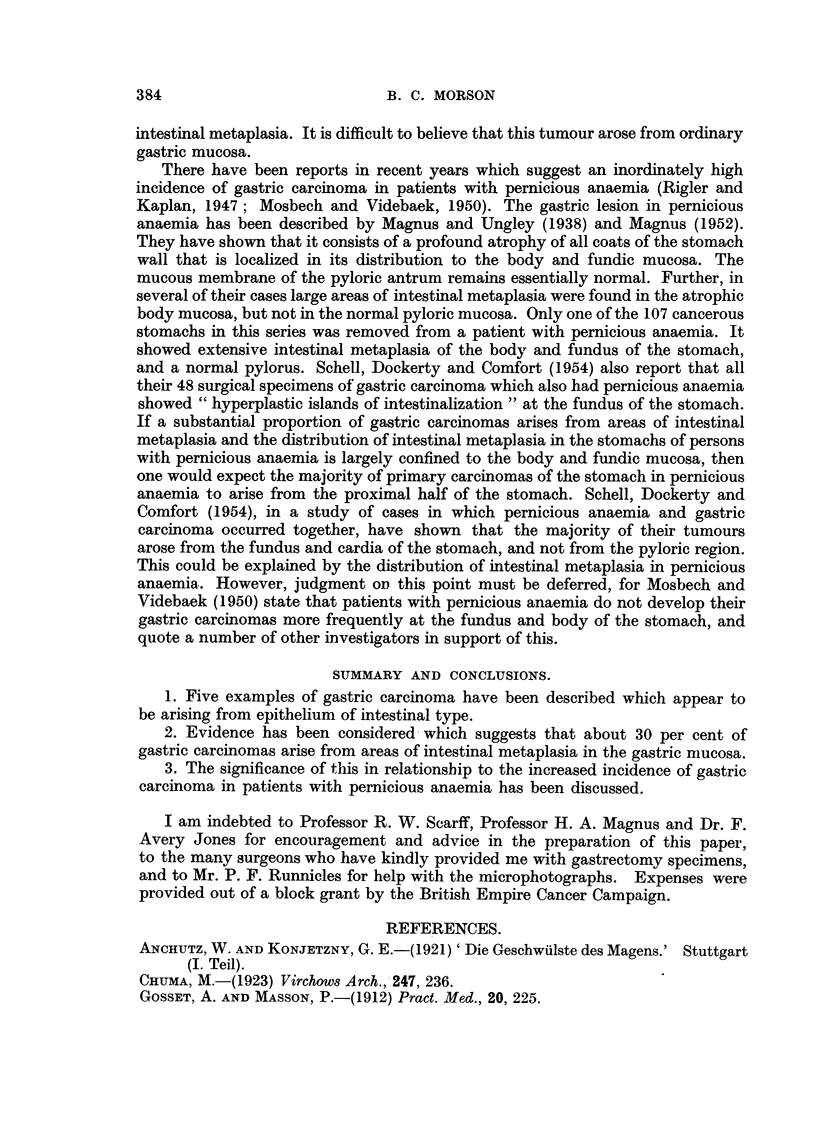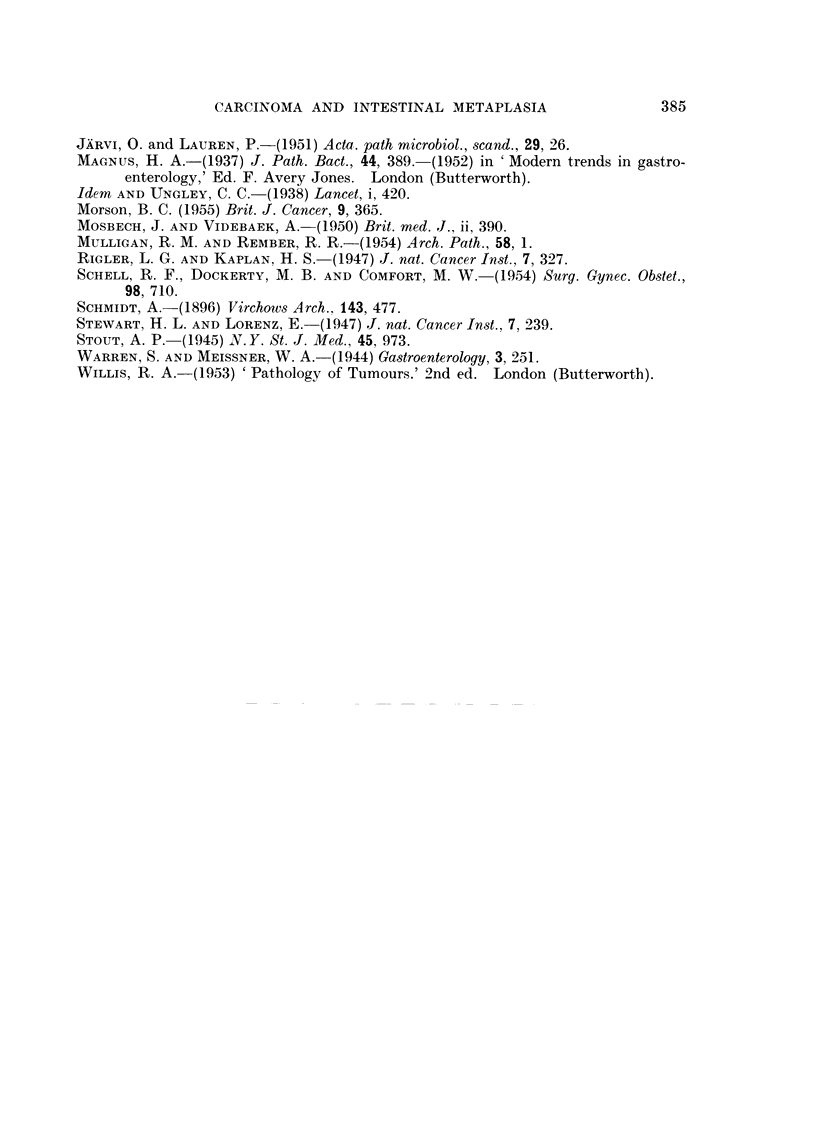# Carcinoma Arising from Areas of Intestinal Metaplasia in the Gastric Mucosa

**DOI:** 10.1038/bjc.1955.36

**Published:** 1955-09

**Authors:** B. C. Morson

## Abstract

**Images:**


### CARCINOMA ARISING FROM AREAS OF INTESTINAL METAPLASIA IN THE GASTRIC MUCOSA

B. C. MORSON.

*From the Cancer Research Department, Mount Vernon Hospital, Northwood.*

Received for publication May 26, 1955.

IT has been demonstrated that there is more intestinal metaplasia of the gastric mucosa in cancerous than non-cancerous stomachs (Stout, 1945 : Morson, 1955). Also, that very large areas of the gastric mucous membrane may be replaced by epithelium of intestinal type. In view of these findings it is suggested that some cases of gastric carcinoma may arise from areas of intestinal metaplasia. Histological evidence in support of this is presented in the following pages.

### MATERIAL AND METHODS.

1. The primary growth and the mucous membrane in its vicinity have been examined in 107 gastrectomy specimens removed for carcinoma of the stomach. Every one of these specimens had at least 2 and often as many as 6 blocks of tissue taken from the primary growth. All of them were cut from the edge of the primary carcinoma and include a strip of the adjacent mucous membrane. In this way any transition from metaplastic mucosa to carcinoma may be detected.

2. In addition to pieces of tissue from the primary growth " swiss roll " sections of the gastric mucosa (Magnus, 1937 ; Morson, 1955) were available in the search for areas of early malignant change. The appearances of pre-invasive carcinoma or carcinoma *in situ* are valuable evidence of the histogenesis of malignant tumours. The absence of invasion answers the criticism that the appearances may have been produced by infiltration of the mucous membrane from without.

All sections were stained with haematoxylin and eosin, and with Southgates' modification of Mayers' muci-carmine for confirming the presence of intestinal mucus.

### RESULTS.

Five examples of the origin of gastric carcinoma from areas of intestinal metaplasia are described and illustrated with microphotographs. In the first of these a gastrectomy specimen is described which contains a very early carcinoma apparently arising from an intestinal type of epithelium. In Examples 2 and 3 the transition from metaplastic mucosa to carcinoma at the edge of a primary tumour is demonstrated. In neither of these cases is there any invasion of the submucosa at the point of transition, and the histological appearances do not suggest invasion of the mucous membrane from without. In the last two examples the appearances of pre-invasive carcinoma or carcinoma *in situ* arising in epithelium of intestinal type are described. These two examples were found in gastrectomy specimens removed for carcinoma, but at a distance from the main tumour. They may be regarded as independent, primary foci of malignancy. There was no haematoxylin and red with muci-carmine. This suggests an origin from an intestinal type of epithelium. Sections taken from the edge of the tumour show complete replacement of the adjacent mucosa by an epithelium of intestinal type (Fig. 4). There is also a transition from metaplastic mucosa to carcinoma at this point. As there is no invasion of the muscularis mucosae or the submucosa where the transition takes place it is justifiable to assume that the malignant change is occurring in the metaplastic mucosa and the appearances are not those of invasion from without.

A study of the histological appearances at the point of transition reveals the following changes (Fig. 5 and 6). Passing from the metaplastic mucosa into carcinoma the tubules lose their regular outline. The cells lining them have become reduplicated and their nuclei are large and hyperchromatic. Many mitoses may be seen. At many points the hyperplastic cells have broken down the limiting membrane of the tubules and are invading the stroma. In other words the tissue has all the characteristics of malignant transformation. At the same time it contains the characteristics of an intestinal type of epithelium. Numerous goblet cells are present and occasional Paneth cells may be seen at the bases of the tubules. Moreover, there is a complete absence of any of the features of ordinary gastric mucosa.

In view of the extensive replacement of the lining of this stomach by epithelium of intestinal type, the nature of the mucus secreted by the tumour, and the histological evidence of a transition from metaplastic mucosa to carcinoma at the edge of the primary growth, it would appear that this carcinoma is arising from an area of intestinal metaplasia and not from ordinary gastric mucosa.

*Example No.* 3.

*Description of specimen.*—Partial gastrectomy with attached greater omentum. There is a nodular growth involving the entire circumference of the pylorus for a length of 1 inch. It extends right up to the pyloro-duodenal junction, but there is no invasion of the duodenum. No other macroscopic abnormality seen in the stomach.

*Histology.* (Fig. 7 and 8).—Sections show a well-differentiated adenocarcinoma invading the stomach wall and peri-gastric tissues. It is secreting a little mucus. Strips of mucous membrane from the region of the pylorus and body of the stomach show extensive areas of intestinal metaplasia.

This tumour is indistinguishable from those found in the large intestine and rectum. It is secreting mucus which stains blue with Ehrlich's haematoxylin and red with muci-carmine. This suggests an origin from an intestinal type of epithelium. Sections taken from the edge of the tumour show complete replacement of the adjacent mucous membrane by epithelium of intestinal type. At this point there is a transition from metaplastic mucosa to carcinoma (Fig. 7). As there is no invasion of the submucosa where the transition takes place it can be presumed that the appearances are those of malignant change *in situ* and not invasion from without.

A study of the histological appearances at the point of transition in this case reveals the following changes (Fig. 8). Passing from the metaplastic mucosa into carcinoma there is a gradual loss of differentiation. The tubules have become distorted in shape and size, and many of them are solid with proliferating cells.

Others are disintegrating and their cells are invading the surrounding stroma. The cells lining the tubules contain large, hyperchromatic nuclei and many mitoses can be seen. These are the characteristics of malignant change. The microphotographs (Fig. 7 and 8) show the likeness between the metaplastic mucosa and the carcinoma. Further, there appears to be a gradual transition from the one to the other. The metaplastic mucosa contains numerous goblet cells which gradually disappear as one passes further into the carcinomatous tissue. A number of Paneth cells are present. There is very little evidence of the presence of any ordinary gastric mucosa.

This stomach shows extensive replacement of its mucosa by an intestinal type of epithelium and its carcinoma is secreting mucus of intestinal type. Furthermore, there is a transition from metaplastic mucosa to carcinoma at the edge of the primary growth. In view of these findings it would appear that this carcinoma is arising from an area of intestinal metaplasia in the gastric mucosa.

---

### EXPLANATION OF PLATES.

FIG. 1.—Example 1. Early adenocarcinoma of the stomach with invasion of submucous tissues. Haematoxylin and eosin. × 10.

FIG. 2.—Example 1. A higher power view of the junction between carcinoma and metaplastic mucosa at the extreme right-hand edge of the previous figure. Note the numerous goblet cells in the metaplastic mucosa. No gastric-type epithelium can be seen. Haematoxylin and eosin. × 50.

FIG. 3.—Example 1. Intestinal type of mucosa in neighbourhood of tumour. Note goblet cells. At the bases of the tubules there are numerous Paneth cells which appear very dark in the photograph. No normal gastric mucosa present. Haematoxylin and eosin. × 50.

FIG. 4.—Example 2. Junction of carcinoma with surrounding mucous membrane. Metaplastic mucosa on right. Note numerous goblet cells and absence of ordinary gastric mucosa. Invasive carcinoma at bottom left-hand corner of the photograph. There appears to be a transition from metaplastic mucosa to carcinoma. Haematoxylin and eosin. × 50.

FIG. 5.—Example 2. Higher power view of long tubule at left centre of Fig. 4. The appearances are those of an intestinal type of epithelium undergoing malignant change. Haematoxylin and eosin. × 100.

FIG. 6.—Example 2. High power view of left centre of previous figure. The characteristics of carcinoma are superimposed upon an intestinal type of epithelium containing numerous goblet cells. Haematoxylin and eosin. × 400.

FIG. 7.—Example 3. Junction of metaplastic mucosa and carcinoma at the edge of the primary tumour. There is continuity between the benign and malignant tissue. Invasion of submucosa at bottom left-hand corner. Haematoxylin and eosin. × 50.

FIG. 8.—Example 3. Higher power view of lower limit of previous figure. The metaplastic mucosa on the right shows numerous goblet cells and absence of characteristic gastric-type epithelium. Passing to the left of the photograph the metaplastic mucosa appears to be undergoing malignant transformation. Haematoxylin and eosin. × 100.

FIG. 9.—Example 4. Carcinoma *in situ*. The characteristics of malignant change are superimposed upon epithelium of intestinal type. No normal gastric mucosa is present. Haematoxylin and eosin. × 100.

FIG. 10.—Example 4. High power view of bottom right-hand corner of previous photograph. Note goblet cells and obvious carcinoma with early invasion of the stroma. Haematoxylin and eosin. × 450.

FIG. 11.—Example 4. Another view of area of carcinoma *in situ*. There is no invasion of the submucosa and the characteristics of intestinal epithelium and carcinoma are present. Haematoxylin and eosin. × 100.

FIG. 12.—Example 5. Carcinoma *in situ*. Numerous goblet cells are present, and the tubules are of intestinal type. The tubule at the left lower margin of the photograph is reminiscent of a normal pyloric gland. At a number of points the metaplastic tubules appear to be undergoing malignant change with invasion of the mucosal stroma. Haematoxylin and eosin. × 100.

FIG. 13.—Example 5. High power view of left centre of previous figure. Tubules lined by goblet cell epithelium are disintegrating and invading the surrounding stroma. Haematoxylin and eosin. × 450.

1

2

3

Morson.

4

5

6

Morson.

7

8

Morson.

11

10

9

Morson.

12

13

*Example No.* 4. (Fig. 9, 10, and 11).

During the examination of a " swiss roll " section of gastric mucosa from the posterior wall of one of the stomachs in this series an example of pre-invasive carcinoma was found. This appears to be arising in epithelium of intestinal type without invasion of the submucous tissues. It is involving a strip of mucous membrane about $\frac{1}{2}$–1 inch long and is surrounded by mucosa which shows complete intestinal metaplasia. Little evidence of normal gastric mucosa can be seen, and there is a complete absence of characteristic gastric glands.

This area of pre-invasive carcinoma shows the features of intestinal epithelium as well as those of carcinoma. The tubules contain numerous goblet cells, which give the characteristic staining reactions for intestinal as opposed to gastric mucus. They are lined by cells containing large, hyperchromatic nuclei (Fig. 10) which show great variation in size and shape. Numerous mitoses are present. The tubules appear to be losing their differentiation, and are disintegrating to form a mass of carcinomatous cells (Fig. 9 and 11) which are invading the mucosal stroma (Fig. 10). There is no sign whatever of any ordinary gastric mucosa, and the malignant change appears to be superimposed upon a mucosa with the characteristics of an intestinal type of epithelium.

*Example No.* 5. (Fig. 12 and 13).

The examination of a " swiss roll " section containing a long strip of pyloric mucosa shows an area of intestinal metaplasia in which there are scattered foci of malignant change. There is hardly any evidence of the presence of ordinary gastric mucous membrane and the histological appearances suggest that the malignant change is superimposed upon epithelium of intestinal type.

The tubules are lines by columnar epithelium containing numerous goblet cells, which give the staining reactions for intestinal mucus. At the bases of the tubules the epithelium is very hyperplastic and many Paneth cells are present. Some of the tubules appear to be disintegrating, with loss of differentiation and invasion of the mucosal stroma by malignant cells. Numerous mitoses are present, some of them abnormal. A study of Fig. 12 gives a representative picture of this area of pre-invasive carcinoma. Relatively normal tubules are present which are of the intestinal type. They are mixed up with others which are definitely carcinomatous. In Fig. 13 a high power view is given which confirms the presence of goblet cells and invasion of the mucosal stroma by malignant cells. In Fig. 12 ragged mucous cysts can be seen. These are very commonly present when malignant change is taking place in an area of intestinal metaplasia, and they are probably due to the blocking of tubules by proliferating cells.

### DISCUSSION.

Histologists of the early part of this century (Schmidt, 1896; Gossett and Masson, 1912; Anchutz and Konjetzny, 1921; Chuma, 1923) believed that some cases of gastric carcinoma arise from intestinal epithelium, and the evidence submitted in this paper supports their views. Stout (1945) failed to demonstrate the histological transition from intestinal metaplasia to carcinoma. But Warren and Meissner (1944) suggest that when the epithelial changes in intestinal metaplasia become severe they compare favourably with recognized pre-cancerous conditions found elsewhere in the body.

The evidence submitted in this paper is concerned only with the histological transition from intestinal metaplasia to carcinoma. There does not appear to be any investigation in the literature with which this evidence can be compared. However, it is interesting to contrast the histological appearances with those described and illustrated by Stewart and Lorenz (1947) in their article on the development of carcinoma from intestinal epithelium in mice treated with carcinogenic substances. This article, which is beautifully illustrated with microphotographs, gives the stages in the development of carcinoma from the epithelium lining the small intestine. The appearances are very similar to those described in this series for the transition from intestinal metaplasia of the gastric mucosa to carcinoma. But there is other evidence which supports the conclusion that gastric carcinoma may arise from epithelium of intestinal type.

There is an histological resemblance between gastric and intestinal carcinoma (Gossett and Masson, 1912). The majority of cases in both groups are adenocarcinomas of varying degrees of differentiation. In general, however, gastric cancers tend to be less well-differentiated than their counterparts in the intestine and rectum. There is also a much higher proportion of anaplastic malignant tumours among the gastric carcinomas. The similarity of gastric and intestinal cancer has led Mulligan and Rember (1954) to use the term " intestinal cell " to describe one of their three main histological types of gastric carcinoma. They classify carcinoma of the stomach into " mucous cell", " pyloro-cardiac " cell, and " intestinal cell " types. Out of their total of 138 cases they put 35, or about 25 per cent, in the " intestinal cell " group and trace their origin to areas of intestinal metaplasia in the gastric mucosa.

If some cases of gastric carcinoma arise from areas of intestinal metaplasia then they should contain evidence of the characteristics of intestinal epithelium. These include the secretion of goblet cell mucus and the presence of a striated border to the columnar cells lining the tubules. Neither of these characteristics is found in normal gastric mucosa and are only seen in the stomach when intestinal metaplasia is present. Järvi and Lauren (1951) investigated 184 specimens of gastric carcinoma and found histological evidence of a striated border to the carcinomatous cells in about 50 per cent of cases, even in the metastases. They reject previous theories that this is due to metaplasia within the tumour itself, and suggest that all gastric carcinomas which contain a striated border arise from areas of intestinal metaplasia in the gastric mucosa. They also investigated the characteristics of mucus in gastric cancers, and point out the difference between mucus of characteristically gastric type and that of intestinal type. In 30 per cent of their cases the mucus in the carcinomas was stained exclusively by muci-carmine, and is therefore of intestinal type. They also showed that in tumours containing a striated border, a mucous secretion of intestinal type was mostly observed. Järvi and Lauren (1951) conclude that a substantial proportion of gastric tumours originate from intestinal epithelium. It is a common observation to see mucus secretion in carcinomas of the stomach and Chuma (1923) suggests that the " signet ring " cells in colloid carcinomas are the malignant counterpart of the goblet cells seen in areas of intestinal metaplasia.

It is important to know what porportion of gastric carcinomas arise from areas of intestinal metaplasia. Of the 107 primary carcinomas examined in this study 35, or 32·7 per cent appear to be arising from epithelium of intestinal type. However, in some cases of carcinoma in this series it was evident that the tumour was not arising from intestinal epithelium. A small proportion of the stomachs containing carcinoma showed no intestinal metaplasia at all. In others very little was seen. These points are mentioned because it is apparent that although a substantial proportion of gastric carcinomas may arise from areas of intestinal metaplasia, in many cases the malignant change occurs in other types of epithelium. It is conceivable that a carcinoma may arise from a solitary area of intestinal metaplasia which is completely destroyed by the expanding tumour. However, in the great majority of cases in which the origin of the primary growth from intestinal epithelium could be demonstrated there was very extensive intestinal metaplasia of the rest of the gastric mucosa. Mulligan and Rember (1954), place about 25 per cent of gastric carcinomas in their " intestinal cell " group. Järvi and Lauren (1951) have shown that about 50 per cent of gastric carcinomas contain evidence of a striated border to their cells, and about 30 per cent secrete mucus of intestinal type. When these figures are taken into account an estimate that about 30 per cent of gastric carcinomas arise from areas of intestinal metaplasia appears to be reasonable.

The topographical distribution of intestinal metaplasia and primary carcinoma in the stomach is similar. In stomachs removed for duodenal ulcer, gastric ulcer and carcinoma it is always the pylorus that is revealed as the site most frequently and extensively affected by intestinal metaplasia (Morson, 1955). It is also true that primary carcinoma of the stomach is most frequently found at the pylorus. According to Willis (1953) about half of all gastric carcinomas occur at this site. In the series considered in this study 45 per cent arose from the pyloric part of the stomach, 22 per cent from the lesser curvature, and 18 per cent from the region of the cardia. Only one carcinoma was involving the fundus, and three occupied the region of the greater curve. The remainder (11 out of 107 cases) involved more than half the whole stomach. This order of frequency is similar to the distribution of intestinal metaplasia. It has been shown that the incidence and extent of intestinal metaplasia decreases in the order: pylorus, lesser curve, greater curve and fundus. The similarity in the distribution can be further compared when it is remembered that the gastric canal is the part of the stomach most frequently and extensively affected by intestinal metaplasia (Morson, 1955) and accounts for nearly 70 per cent (excluding the cardia) of all cases of gastric carcinoma. If some cases of carcinoma arise from areas of intestinal metaplasia then one would expect the distribution of these two conditions to be similar.

It has been shown that nearly 80 per cent of a series of 119 stomachs removed for duodenal ulcer, gastric ulcer and carcinoma contain areas of intestinal metaplasia (Morson, 1955). In many of these the gastric mucosa was very extensively replaced by epithelium of intestinal type. Further, in most of the cases of carcinoma in which the transition from intestinal metaplasia to carcinoma can be seen the surrounding mucous membrane showed complete intestinal metaplasia. It would not be surprising from these facts alone if some cases of gastric carcinoma arise from epithelium of intestinal type. It is always easier to demonstrate the type of tissue from which a growth is arising by the study of very early carcinomas. In these the type of mucous membrane adjacent to the tumour gives evidence of its histogenesis. In Example No. 1, the primary carcinoma is a very small one and is only just beginning to invade the stomach wall. The mucous membrane around it (Fig. 3) shows complete metaplasia to an intestinal type of epithelium. In fact, almost the whole of the stomach lining in this case shows intestinal metaplasia. It is difficult to believe that this tumour arose from ordinary gastric mucosa.

There have been reports in recent years which suggest an inordinately high incidence of gastric carcinoma in patients with pernicious anaemia (Rigler and Kaplan, 1947 ; Mosbech and Videbaek, 1950). The gastric lesion in pernicious anaemia has been described by Magnus and Ungley (1938) and Magnus (1952). They have shown that it consists of a profound atrophy of all coats of the stomach wall that is localized in its distribution to the body and fundic mucosa. The mucous membrane of the pyloric antrum remains essentially normal. Further, in several of their cases large areas of intestinal metaplasia were found in the atrophic body mucosa, but not in the normal pyloric mucosa. Only one of the 107 cancerous stomachs in this series was removed from a patient with pernicious anaemia. It showed extensive intestinal metaplasia of the body and fundus of the stomach, and a normal pylorus. Schell, Dockerty and Comfort (1954) also report that all their 48 surgical specimens of gastric carcinoma which also had pernicious anaemia showed " hyperplastic islands of intestinalization " at the fundus of the stomach. If a substantial proportion of gastric carcinomas arises from areas of intestinal metaplasia and the distribution of intestinal metaplasia in the stomachs of persons with pernicious anaemia is largely confined to the body and fundic mucosa, then one would expect the majority of primary carcinomas of the stomach in pernicious anaemia to arise from the proximal half of the stomach. Schell, Dockerty and Comfort (1954), in a study of cases in which pernicious anaemia and gastric carcinoma occurred together, have shown that the majority of their tumours arose from the fundus and cardia of the stomach, and not from the pyloric region. This could be explained by the distribution of intestinal metaplasia in pernicious anaemia. However, judgment on this point must be deferred, for Mosbech and Videbaek (1950) state that patients with pernicious anaemia do not develop their gastric carcinomas more frequently at the fundus and body of the stomach, and quote a number of other investigators in support of this.

### SUMMARY AND CONCLUSIONS.

1. Five examples of gastric carcinoma have been described which appear to be arising from epithelium of intestinal type.

2. Evidence has been considered which suggests that about 30 per cent of gastric carcinomas arise from areas of intestinal metaplasia in the gastric mucosa.

3. The significance of this in relationship to the increased incidence of gastric carcinoma in patients with pernicious anaemia has been discussed.

I am indebted to Professor R. W. Scarff, Professor H. A. Magnus and Dr. F. Avery Jones for encouragement and advice in the preparation of this paper, to the many surgeons who have kindly provided me with gastrectomy specimens, and to Mr. P. F. Runnicles for help with the microphotographs. Expenses were provided out of a block grant by the British Empire Cancer Campaign.

### REFERENCES.

ANCHUTZ, W. AND KONJETZNY, G. E.—(1921) ' Die Geschwülste des Magens.' Stuttgart (I. Teil).

CHUMA, M.—(1923) *Virchows Arch.*, **247**, 236.

GOSSET, A. AND MASSON, P.—(1912) *Pract. Med.*, **20**, 225.

JÄRVI, O. and LAUREN, P.—(1951) *Acta. path microbiol., scand.*, **29**, 26.
MAGNUS, H. A.—(1937) *J. Path. Bact.*, **44**, 389.—(1952) in ' Modern trends in gastroenterology,' Ed. F. Avery Jones. London (Butterworth).
*Idem* AND UNGLEY, C. C.—(1938) *Lancet*, i, 420.
Morson, B. C. (1955) *Brit. J. Cancer*, **9**, 365.
MOSBECH, J. AND VIDEBAEK, A.—(1950) *Brit. med. J.*, ii, 390.
MULLIGAN, R. M. AND REMBER, R. R.—(1954) *Arch. Path.*, **58**, 1.
RIGLER, L. G. AND KAPLAN, H. S.—(1947) *J. nat. Cancer Inst.*, **7**, 327.
SCHELL, R. F., DOCKERTY, M. B. AND COMFORT, M. W.—(1954) *Surg. Gynec. Obstet.*, **98**, 710.
SCHMIDT, A.—(1896) *Virchows Arch.*, **143**, 477.
STEWART, H. L. AND LORENZ, E.—(1947) *J. nat. Cancer Inst.*, **7**, 239.
STOUT, A. P.—(1945) *N.Y. St. J. Med.*, **45**, 973.
WARREN, S. AND MEISSNER, W. A.—(1944) *Gastroenterology*, **3**, 251.
WILLIS, R. A.—(1953) ' Pathology of Tumours.' 2nd ed. London (Butterworth).